# Anaphylaxis triggers in a large tertiary care hospital in Qatar: a retrospective study

**DOI:** 10.1186/s40413-018-0200-9

**Published:** 2018-09-04

**Authors:** Taghreed Abunada, Maryam Ali Al-Nesf, Lukman Thalib, Rana Kurdi, Sally Khalil, Wessam ElKassem, Hassan M. Mobayed, Hatem Zayed

**Affiliations:** 1 Biomedical Science Department, College of Health Science, Qatar University, P.O. Box 2713, Doha, Qatar; 2 Allergy and Immunology Unit, Hamad Medical Corporation, P.O. Box 3050, Doha, Qatar; 3 College of Health Science, Qatar University, P.O. Box 2713, Doha, Qatar; 4 Department, College of Health Science, Qatar University, P.O. Box 2713, Doha, Qatar; 5 Allergy & Clinical Immunology Unit, Hamad Medical Corporation, P.O. Box 3050, Doha, Qatar; 6 Pharmacy Department, Women’s Hospital, Hamad Medical Corporation, P.O. Box 3050, Doha, Qatar; 7 Hamad Medical Corporation, P.O. Box 3050, Doha, Qatar

**Keywords:** Anaphylaxis, Allergy, Triggers, Qatar

## Abstract

**Background:**

Anaphylaxis is a serious allergic disease that may lead to death if not immediately recognized and treated. Triggers of anaphylaxis including food, drugs, and insect stings can vary widely. The incidence of anaphylaxis seems to be affected by age, sex, atopy, and geographic location. This study aims to examine the common triggers of anaphylaxis in Qatar.

**Methods:**

A total of 1068 electronic medical records were audited using power chart system: 446 from the medical coding system of anaphylaxis and 622 from the epinephrine auto-injectors (EAIs) dispensed during January 2012–December 2017.

**Results:**

Of 1068 patients, 574 (53.5%) had anaphylaxis; male to female ratio was 1.2, and 300 patients (77.9%) were less than 10 years old. The common triggers were food (*n* = 316, 55.0%), insect stings (*n* = 161, 28.0%), and drugs (*n* = 103, 17.9%). Common anaphylaxis food triggers were nuts (*n* = 173, 30.1%), eggs (*n* = 89, 15.5%), and seafood (*n* = 72, 12.5%), and common anaphylaxis medication triggers were antibiotics (*n* = 49, 8.5%) and nonsteroidal anti-inflammatory drugs (*n* = 30, 5.2%). Interestingly, 135 anaphylactic patients (23.5%) were due to black ant stings. The anaphylaxis triggers varied significantly between children and adults. Among children (less than 10 years), three quarters of the events were triggered by food (223, 74.3%) while among adults (20–55 years), insect stings (*n* = 59, 43.0%) and drugs (*n* = 44, 32.0%) were dominant.

**Discussion:**

This is the first national study stratifying anaphylaxis triggers among different age groups in Qatar. This study will serve as a guide for clinical practice in allergy clinics in Qatar and will help to assess future trends of anaphylaxis in Qatar.

**Electronic supplementary material:**

The online version of this article (10.1186/s40413-018-0200-9) contains supplementary material, which is available to authorized users.

## Background

Anaphylaxis is a serious systemic allergic reaction that is rapid in onset and may be fatal if not immediately recognized and treated [1–3]. Triggers of anaphylaxis vary widely and include food, drugs, and insect stings. Once triggered, the disease manifests itself by compromising the function of multiple organs, including skin (90%), respiratory (70%), gastrointestinal (30–45%), cardiovascular (35%) and central nervous system (10–15%) [2, 4]. Personal predisposition and family history of atopy usually worsen the course of anaphylaxis in affected subjects [2–4].

Although it is difficult to characterize anaphylaxis incidence due to its transient acute nature and under-recognition especially in case of cutaneous symptoms absence (20% of the cases) [3, 5, 6]. Several studies from USA, UK, and Australia suggested that the incidence of anaphylaxis is on a gradual rise over the last two decades [7–14]. To estimate the incidence, prevalence, and triggers, scientists have used different methodologies including patients’ case reports [15–17], international medical coding systems [7–9, 18–24], hospital admission rates [11, 13, 25, 26], public surveys and epinephrine dispense records [10, 12, 21, 25, 27, 28]. These studies have demonstrated that distribution of anaphylaxis tends to fluctuate based on age, gender, race, geographical residence, and socioeconomic status of involved subjects.

Anaphylaxis was described in a few case-reports in Qatar [15, 29–31], however, its triggers have not been thoroughly studied. The aim of this study is to retrospectively estimate and describe the distribution of anaphylaxis triggers in different age and gender groups in Qatar from January 2012 to December 2016.

## Method

### Data collection

**Fig. 1:**
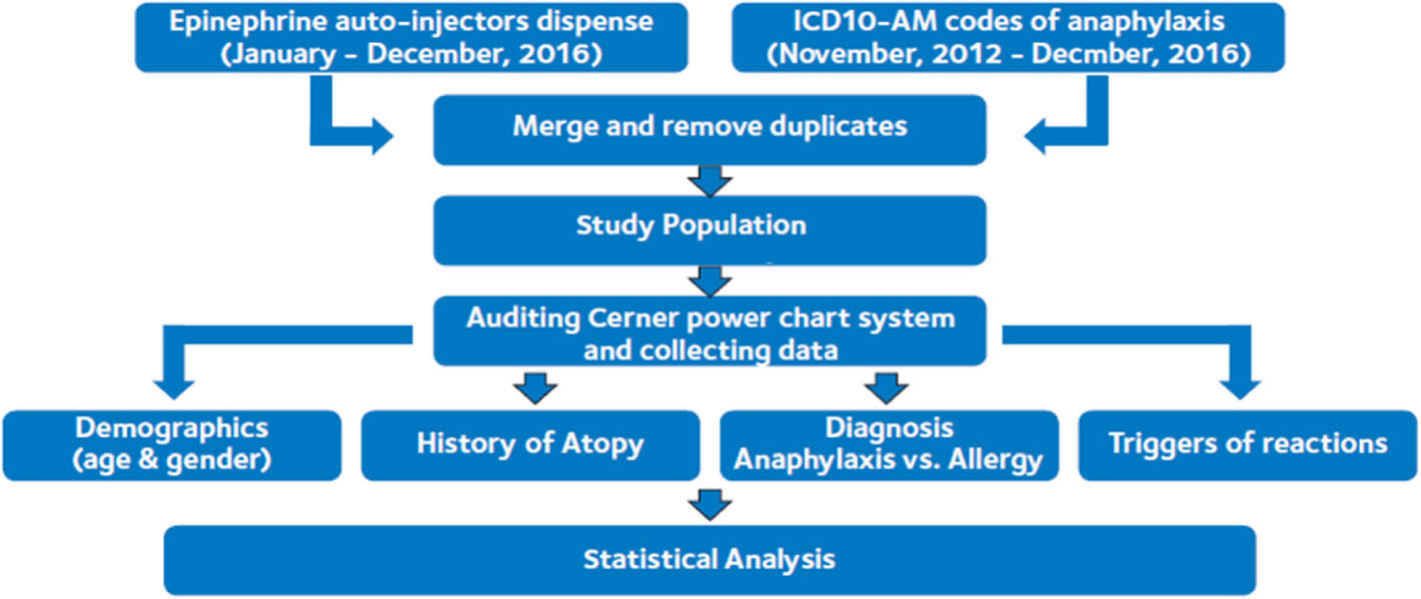
Flowchart of the study design

Between January 2012–December 2016, electronic medical records were reviewed retrospectively using Cerner power chart system. This includes patients admitted and registered in Cerner power chart system with the International Classification of Diseases 10th revision-Australian Modification (ICD10-AM) and discharged with diagnostic codes of anaphylaxis: T 78.0 (anaphylactic shock due to adverse food reactions), T 78.1 (other adverse food reactions, not elsewhere classified), T78.2 (anaphylactic shock, unspecified), T80.5 (anaphylactic shock due to serum), or T88.6 (anaphylactic shock due to adverse effect of correct drug or medication properly administered) and patients who had Epinephrine Auto-Injector (EAIs) dispensed from Hamad General Hospital pharmacy (Fig. 1).

### Sample selection

The study was approved by Hamad Medical Corporation (HMC) local ethics committee (IRB 17122/17). Anaphylaxis was defined based on physician diagnosis and the clinical protocols of HMC that are in accordance with the clinical criteria of anaphylaxis guidelines [32]. Our inclusion criteria patients with anaphylaxis were either one of the following: (1) acute onset of illness (minutes to several hours) with involvement of the skin, mucosal tissue or both, and at least respiratory compromise or reduced blood pressure; (2) involvement of two or more: skin-mucosal, respiratory, gastrointestinal and/ or hypotension (minutes to several hours) after exposure to a likely allergen; or (3) reduced blood pressure after exposure to a known allergen (minutes to several hours). Generalized Allergic Reaction (GAR) was identified as patients who were exposed to triggers that resulted in symptoms of allergic reaction without fulfilling the clinical criteria of anaphylaxis. Patients with GAR may have underlying allergic diseases such as asthma, atopic dermatitis, urticaria, angioedema or allergic rhinitis. Anaphylaxis and GAR definitions were applied to the records that were reviewed**.** Demographic information and clinical diagnosis of patients were retrieved, reviewed, and documented anonymously, and then sub-categorized to be analyzed based on gender, age, history of atopy, symptoms, and triggers.

### Triggers

Triggers were defined as etiological agents that may lead to either GAR or anaphylaxis [1, 3] . Triggers were classified into food, drugs, insect stings, or idiopathic factors. All the triggers of allergic reactions and anaphylaxis were identified based on patient’s history of exposure to the triggers and the circumstances accompanying the reactions that have been recognized and confirmed by the treating physician. These details were documented by the treating physicians in the electronic medical records. When possible triggers of the reactions were not clearly recognized by the patients or physicians, they were classified to be idiopathic.

### Statistical analysis

Data analysis was performed using Statistical Package for Social Sciences (SPSS Chicago IL, USA). Groups were compared using chi-square test and the Fisher’s exact test (two-tailed) replaced the chi-square in case of small sample size, where the expected frequency is less than 5 in any of the cells. The level where *P* <  0.05 (two-tailed) was considered as the cut-off for significance.

## Results

### Characteristics of the study population

Out of 1068 electronic medical records audited using Cerner power chart system; 446 inpatients registered with ICD-10 codes of anaphylaxis and 622 outpatients had EAIs dispensed. Five hundred seventy-four patients (53.5%) had anaphylaxis; 315 (54.8%) were males and 300 (52.2%) were children less than 10 years old, 251 patients (43.7%) were Qatari, 162 patients (28.2%) were non-Qatari Arabs, and 118 patients (20.5%) were Asian. Personal history of asthma, atopic dermatitis, urticaria and allergic rhinitis were determined in 208 (36.2%), 195 (33.9%), 179 (31.1%), and 81 (14.1%) respectively. One-fifth of the study population had a positive family history of atopy (Table 1).

**Table 1:** Characteristics of the study population

Characteristic	Total *N* = 1068 *n* (%)	Anaphylaxis *N* = 574 *n* (%)	GAR *N* = 132 *n* (%)	*P*-value
Age (Years)				
< 10	603 (56.3)	300 (52.2)	85 (64.3)	0.009^a^
10–19	210 (19.7)	109 (18.9)	22 (16.6)	
20–55	209 (19.6)	137 (23.8)	21 (15.9)	
>. 55	46 (4.3)	28 (4.8)	4 (3.0)	
Gender				
Male	612 (57.3)	315 (54.8)	83 (62.8)	0.095
Female	456 (42.7)	259 (45.2)	49 (37.1)	
Nationality, *N* = 1067^b^				
Qatari	438 (41.0)	251 (43.7)	63 (47.7)	0.009
Non-Qatari, Arab	303 (28.4)	162 (28.2)	25 (18.9)	
Asian	228 (21.4)	118 (20.5)	24 (18.1)	
Others	98 (9.2)	42 (7.3)	20 (15.1)	
Personal History				
Asthma	357 (36.4)	208 (36.2)	68 (51.5)	< 0.001
Atopic dermatitis	326 (33.2)	195 (33.9)	66 (50)	< 0.001
Urticaria/ angioedema	254 (25.9)	179 (31.1)	36 (27.2)	0.485
Allergic rhinitis	142 (14.5)	81 (14.1)	30 (22.7)	0.009
Family History, *N* = 123				
Atopy ^c^	70 (56.9)	58 (10.1)	8 (6.1)	0.989
Anaphylaxis	6 (4.9)	5 (0.8)	1 (0.7)	
Consanguinity, *N* = 33	30 (90.9)	25 (4.3)	2 (1.5)	1.000^*^

*GAR* generalized allergic reactions

^a^ Chi-square for trend (linear by linear association)

^b^ One patient had no listed nationality in the system

^c^ Atopy includes asthma, atopic dermatitis, urticaria and allergic rhinitis

*P-value is for Fischer test (exact significant 2-sided)

### Triggers

Overall, triggers were not identified in 44 cases (7.6%) of anaphylaxis and five cases (3.7%) of GAR. Food accounted for 403 (37.7%), followed by insects’ stings 184 (17.2%) and drugs 123 (11.5%). The common triggers of anaphylaxis are detailed in Table 2. Other triggers that contributed to anaphylaxis were cold (3, 0.5%), latex (2, 0.3%), contrast media (2, 0.3%), exercises (1, 0.1%) and food-dependent exercise-induced anaphylaxis (1, 0.1%) (Table 2).

**Table 2:** Causative triggers of symptoms in the study population

Causative triggers	Total *N* = 1068 *n* (%)	Anaphylaxis *N* = 574 *n* (%)	GAR *N* = 132 *n* (%)	*P*-value
Food (All)	403 (37.7)	316 (55.0)	87 (65.9)	< 0.001
Nuts ^a^	232 (21.7)	173 (30.1)	59 (44.6)	< 0.001
Egg	113 (10.5)	89 (15.5)	24 (18.1)	0.171
Seafood	93 (8.7)	72 (12.5)	21 (15.9)	0.111
Peanuts	92 (8.6)	71 (12.3)	21 (15.9)	0.100
Cow’s milk	77 (7.2)	61 (10.6)	16 (12.1)	0.326
Sesame seeds	65 (6.1)	50 (8.7)	15 (11.3)	0.158
Wheat	38 (3.5)	35 (6.1)	3 (2.2)	0.130
Other food ^b^	150 (14.0)	126 (21.9)	24 (18.1)	0.933
Insects’ stings (All)	184 (17.2)	161 (28.0)	23 (17.4)	0.122
Black ant	153 (14.3)	135 (23.5)	18 (13.6)	0.101
Bee	3 (0.2)	3 (0.5)	0 (0.0)	1.000^*^
Wasp	1 (0.1)	1 (0.1)	0 (0.0)	1.000^*^
Unspecified	29 (2.7)	24 (4.1)	5 (3.7)	0.798^*^
Drugs (All)	123 (11.5)	103 (17.9)	20 (16.2)	0.978
Antibiotics	58 (5.4)	49 (8.5)	9 (6.8)	0.883
Augmentin	19 (1.7)	16 (2.7)	3 (2.2)	1.000^*^
Penicillin	14 (1.3)	11 (1.9)	3 (2.2)	0.484^*^
Ceftriaxone	6 (0.5)	6 (1.0)	0 (0.0)	0.596^*^
Amoxicillin	6 (0.5)	5 (0.8)	1 (0.7)	1.000^*^
Other antibiotics	22 (2.0)	19 (3.3)	3 (2.2)	1.000^*^
NSAID ^c^	36 (3.3)	30 (5.2)	6 (4.5)	0.938
Ibuprofen	28 (2.6)	23 (4.0)	5 (3.7)	0.794^*^
Paracetamol	8 (0.7)	8 (1.3)	0 (0.0)	0.366^*^
Diclofenac	8 (0.7)	7 (1.2)	1 (0.7)	1.000^*^
Aspirin	3 (0.3)	3 (0.5)	0 (0.0)	1.000^*^
Other NSAID	4 (0.3)	4 (0.6)	0 (0.0)	1.000^*^
IvIg ^d^	4 (0.3)	4 (0.6)	0 (0.0)	1.000^*^
Vaccines	3 (0.3)	3 (0.5)	0 (0.0)	1.000^*^
Other drugs	41 (3.8)	35 (6.1)	6 (4.5)	0.779
Idiopathic (All)	49 (4.5)	44 (7.6)	5 (3.7)	0.245

^a^Nuts included cashew, pistachio, tree nuts, coconuts and hazelnuts excluding peanuts which has been counted separately

^b^Other food included chickpeas, bean, lentil, strawberry, banana, kiwi, mango, chicken, beef, pineapple, apple, and watermelon

^c^*NSAID* non-steroidal anti-inflammatory drugs

^d^*IvIg* Intravenous immunoglobulin

*P-value is for Fischer test (exact significant 2-sided)

### Age and gender variation in anaphylaxis

**Fig. 2:**
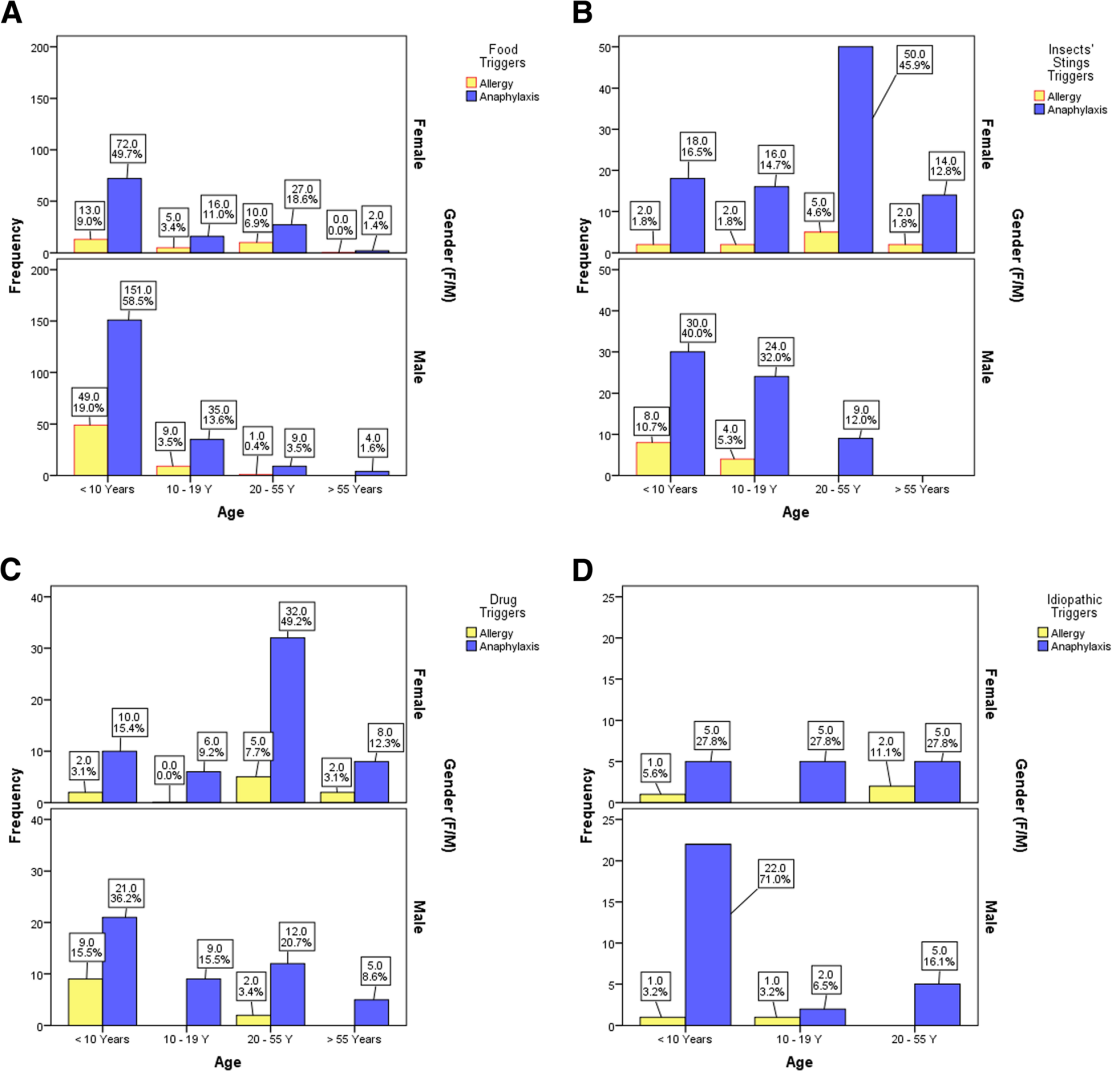
Distribution of anaphylaxis pattern among different age and gender groups. **a** Food triggers, **b** Insect stings triggers, **c** Drug triggers, **d** Idiopathic triggers

Insects’ stings, food, and drug were significantly different between the different age groups (*P* <  0.001), while only the food and insects’ stings showed significance among gender groups (*P* <  0.001). The nationality of patients with anaphylaxis showed no such significant difference in relation to anaphylaxis triggers (Additional file 1: Table S1). The distribution of anaphylaxis and GAR among different age and gender groups is shown in Fig. 2.

## Discussion

This study stratifies anaphylaxis triggers among different age and gender groups and provides a profile of the common allergens that trigger anaphylaxis, to alert clinicians and serve as a baseline to assess future trends of anaphylaxis triggers in Qatar. We were able to identify 574 cases of anaphylaxis out of 1068 records. Food was the leading trigger of anaphylaxis in children regardless of gender. Anaphylaxis induced by drugs and insects’ stings was more common among female adults (Fig. 2). Interestingly, 23.5% of patients had anaphylaxis by black ants.

Our data showed a predominance of anaphylaxis among pediatrics (*n* = 300, 52.2%), which is reasonable since at a single time point anaphylaxis is initially diagnosed at childhood, and relevant triggers avoidance is recommended as preventive measures of a long-term action plan and risk reduction. However, such avoidance measures are neither easily nor strictly followed by children of this age group [16, 17, 33].

Anaphylaxis was common in two age/gender groups: male children (*n* = 224, 39.0%) and female adults (*n* = 114, 19.8%) (Fig. 2), which is consistent with other findings reported by Alshami et al. where they found an incidence of anaphylaxis in pediatric emergency centers of 13.3 per 100,000 visits with 69% of patients being males [34], and Mehdi et al. showed that the incidence among adults was 16.5 per 100,000 visits with 78% being females [35]. Several studies in different ethnic groups showed similar age and gender distribution of anaphylaxis among different age/gender groups; for example, an epidemiological study based on measuring the anaphylaxis rates in emergency department visits in hospitals across Florida, USA, reported that the highest anaphylaxis incidence rate was among the youngest males (8.2/100,000 visits) and the adult females (10.9/100,000 visits) [20]. Similar to this, findings from the Rochester epidemiology project from 1990 through 2000 showed that age-specific incidence rate of anaphylaxis was the highest for ages 0–19 years [8].

In our patients’ cohort, we observed that the association between the development of anaphylaxis compared to GAR and the national origin were statistically significant (*p*-value = 0.009) (Table 1). For instance, “Non-Qatari Arabs” had relatively higher prevalence of anaphylaxis compared to GAR while “Others” such as European, American and African had relatively lower prevalence of anaphylaxis compared to GAR. Such differences in the rates of anaphylaxis compared to GAR associated with ethnic and national groups can be explained by a number of factors, including genetic and environmental exposure. Qatar is a melting pot of hundreds of nationalities of migrant workers [36] that may have different genetic predisposition to allergy and anaphylaxis. In addition to potential differences in the genetic make-up, different life style and dietary patterns as well as differential prevalence of illnesses and use of varying medications may be some of the factors that may or may not contribute to such differences [18, 23]. In general, anaphylaxis was common [7–9, 20, 21], more associated with repeated use of epinephrine [27] and more fatal [23] among Caucasians compared to Black, Latino/Hispanic and Asian ethnicities. In contrast, Mahdavinia et al. (2017) reported that Caucasians had a lower rate of food allergy associated anaphylaxis than African American and Hispanic children [37]. Additionally, Buka et al. (2015) reported that Caucasians had less incidence, and were less likely to present with severe anaphylactic symptoms than South Asian British children living in Birmingham [38].

Personal history of atopy for asthma (*n* = 208, *p* <  0.001), atopic dermatitis (*n* = 195, *p* <  0.001) and allergic rhinitis (*n* = 81, *p* = 0.009) were noted among patients with anaphylaxis in Qatar (Table 1), and 56.9% cases had positive family history (Table 1). Although other studies showed no such significant association between atopy and anaphylaxis [39, 40], atopy was frequently visualized as a risk factor that might worsen the clinical outcome of anaphylaxis [8, 18, 19, 21, 24, 41–43]. However, several studies stated that anaphylaxis was common among patients with atopic diseases. In comparison to our study, different distribution of atopic diseases was observed [8, 18, 19, 21, 24, 41–43].

Our results showed that regardless of gender, food was the culprit for anaphylaxis in children less than 10 years (Fig. 2a). The major causative triggers of food-induced anaphylaxis in Qatar were nuts and eggs, a finding that was consistent with a Saudi finding reported in 2015 [14]. Peanuts, a major trigger of food-related anaphylaxis in the United States [7, 9, 22], is ranked in the fourth position after seafood in Qatar. In a prospective cohort study conducted in Qatar from 2007 to 2010, anaphylaxis induced by cow’s milk proteins (CMP) was found in 10 children out of 38 allergic subjects and suggested camel milk as a safer alternative choice after being experimentally tested [30, 31]. With a larger study population, anaphylaxis induced by cow’s milk was accounted for 61 (10.6%) from 2012 to 2016. In comparison, the prevalence of CMP anaphylaxis resulted in 6–9% of children hospital and emergency admission in the USA [7, 19, 21] and 10% in the UK [44]. CMP anaphylaxis accounted for 8 fatalities in UK children during the period from 1992 to 2012 [13]. Our data showed that sesame seed accounted for 8.7% of anaphylaxis cases in Qatar (Table 2). However, as a global allergen, sesame seed is affecting approximately 0.1% of North American population and is the third common food allergen in Israel [45]. In Lebanon, a cross-sectional study showed that allergic reactions triggered by sesame seed were of severe grade and manifested mainly in the form of anaphylaxis [46]. This study suggested that the sesame seed is the “Middle Eastern” peanut [46].

Anaphylaxis and GAR attributable to Hymenoptera stings in our study demonstrated predominance in female adults (*n* = 50, 45.9%) and male children (*n* = 30, 40.0%) (Fig. 1b). Interestingly, 135 anaphylactic patients (23.5%) developed anaphylaxis by the sting of black ant which is a widespread ant in tropical Africa and the Middle East and is a native insect in Arabian Desert countries, including Qatar [47, 48]. Allergic reactions due to black ant stings range from pain with local itching at the sting site to severe anaphylactic shock. AlAnazi et al. (2009) showed that the diversity of manifestation and human response to black ant stings in four cases encountered in Al Riyadh, the capital city of Saudi Arabia, and three patients were adult females [49]. In contrast to our findings, lower prevalence of black ant induced anaphylaxis was reported in Saudi Arabia (3.2%) [14], and Singapore (12.9%) [50]. The unreported incidence of black Samsum ant induced anaphylaxis was recognized in Iran where most stings result in mild allergic reactions [51]. However, in United Arab Emirates, 4 deaths were recorded after the sting of this ant [52]. Several studies attribute diversity of symptoms to the antigenicity variation of black ants’ toxin composition according to geographical regions [51, 53]. Anaphylaxis in Najran, a city in southwestern Saudi Arabia, was triggered by a different species of black ant, *Solenopsis richteri*, in non-Saudi expatriates (1997–1999) [54]. A Turkish retrospective review defined prevalence of Hymenoptera stings anaphylaxis among adult patients, however, the causative triggers were mainly honey bees and different wasp species [55]. In contrary to Qatar, the later Turkish study showed a predominance of Hymenoptera induced anaphylaxis among male adults (57.1%) [55]. In light of the absence of studies published about black Samsum ant abundance, distribution, and its toxin antigenicity in Qatar, our results flag it as a public health hazard in Qatar owing to its strong association with anaphylaxis.

A key strength of this work includes the fact that Hamad General Hospital, a member of Hamad Medical Corporation, is the only medical facility that dispenses EAIs in Qatar. Therefore, using dispensed (EAI) records of outpatients in combination with medical coding system (ICD-10 AM) of anaphylaxis for inpatients would be an accurate estimation of the prevalence of anaphylaxis in Qatar. Although, EAI dispense records were available for 1 year only (January – December 2016), EAIs as a refilled drug included dispense records of previous years.

## Conclusion

Our study provides new data regarding the frequency of anaphylaxis in our geographical region; however, it is prone to reporting bias due to its retrospective nature and reliance on physician documentation. Besides that, we had 364 medical records (34.1%) with missing data or incomplete charts and we cannot assume them as being negative since there is the possibility of underreporting by physicians. Therefore, the presenting data should be interpreted with caution stating that “***within the boundary of available data”*** registered in Cerner power chart system and out of 1068 subjects, 574 (53.5%) patients had a definite diagnosis of anaphylaxis (2012–2016). Further studies are needed to confirm the medical diagnosis of the missing cases using another method. This study will serve as a platform for clinicians in the allergy clinics in Qatar to improve patient care and for further epidemiological studies for understanding more about the prevalence of anaphylaxis in Qatar. Our data might provide the baseline for assessing future trends. We would recommend integrating entomology, bioecology and medicine points of view to study black ant anaphylaxis in Qatar.

## Additional file

Additional file 1: **Table S1.** Anaphylactic patterns variation in relation to age, gender and nationality. ^a^ row percentage. **Table S2.** Symptoms of the study population. (DOCX 19 kb)

## Abbreviations

CMP: Cow’s milk proteins
EAIs: Epinephrine Auto-injectors
GAR: Generalized Allergic Reaction
HMC: Hamad Medical Corporation
ICD-10 AM: International Classification of Diseases 10th revision-Australian Modification
IVIG: Intravenous Immunoglobulin
NSAID: Non-Steroidal Anti-Inflammatory Drugs
SPSS: Statistical Package for Social Sciences

## References

[1] Keet C (2011). Recognition and management of food-induced anaphylaxis. Pediatr Clin N Am.

[2] Lee JK, Vadas P (2011). Anaphylaxis: mechanisms and management. Clin Exp Allergy.

[3] Simons FE, Ardusso LR, Bilò MB, Cardona V, Ebisawa M, El-Gamal YM (2014). International consensus on (ICON) anaphylaxis. World Allergy Organ J..

[4] Boyce JA, Assa'ad A, Burks AW, Jones SM, Sampson HA, Wood RA (2010). Guidelines for the diagnosis and Management of Food Allergy in the United States: summary of the NIAID-sponsored expert panel report. J Allergy Clin Immunol.

[5] Cox LS, Sanchez-Borges M, Lockey RF (2017). World Allergy Organization Systemic Allergic Reaction Grading System: Is a Modification Needed?. J Allergy Clin Immunol Pract.

[6] Muraro A, Roberts G, Worm M, Bilò MB, Brockow K, Fernández Rivas M (2014). Anaphylaxis: guidelines from the European academy of allergy and clinical immunology. Allergy.

[7] Parlaman JP, Oron AP, Uspal NG, DeJong KN, Tieder JS (2016). Emergency and Hospital Care for Food-Related Anaphylaxis in children. Hosp Pediatr.

[8] Decker WW, Campbell RL, Manivannan V, Luke A, St Sauver JL, Weaver A (2008). The etiology and incidence of anaphylaxis in Rochester, Minnesota: a report from the Rochester epidemiology project. J Allergy Clin Immunol.

[9] Michelson KA, Monuteaux MC, Neuman MI (2016). Variation and trends in anaphylaxis Care in United States Children's hospitals. Acad Emerg Med.

[10] Diwakar L, Cummins C, Ryan R, Marshall T, Roberts T (2017). Prescription rates of adrenaline auto-injectors for children in UK general practice: a retrospective cohort study. Br J Gen Pract.

[11] Gibbison B, Sheikh A, McShane P, Haddow C, Soar J (2012). Anaphylaxis admissions to UK critical care units between 2005 and 2009. Anaesthesia.

[12] Sheikh A, Hippisley-Cox J, Newton J, Fenty J (2008). Trends in national incidence, lifetime prevalence and adrenaline prescribing for anaphylaxis in England. J R Soc Med.

[13] Turner PJ, Gowland MH, Sharma V, Ierodiakonou D, Harper N, Garcez T (2015). Increase in anaphylaxis-related hospitalizations but no increase in fatalities: an analysis of United Kingdom national anaphylaxis data, 1992–2012. J Allergy Clin Immunol.

[14] Sheikh F, Amin R, Rehan Khaliq AM, Al Otaibi T, Al Hashim S, Al GS (2015). First study of pattern of anaphylaxis in a large tertiary care hospital in Saudi Arabia. Asia Pac Allergy.

[15] Mobayed HM, Ali A-NM (2014). Two cases of food-dependent exercise-induced anaphylaxis with different culprit foods. Ann Thorac Med.

[16] Alsalamah M, Makhajia M, Somers G, Marcon M, Hummel D, Upton J (2016). Anaphylaxis to milk after elimination diet for eosinophilic gastrointestinal disease. Am J Gastroenterol.

[17] Zapatero L, Baeza ML, Sierra Z, Molero MI (2005). Anaphylaxis by fruits of the Fagaceae family: acorn and chestnut. Allergy.

[18] Taylor-Black S, Wang J (2012). The prevalence and characteristics of food allergy in urban minority children. Ann Allergy Asthma Immunol.

[19] Huang F, Chawla K, Järvinen KM, Nowak-Węgrzyn A (2012). Anaphylaxis in a New York City pediatric emergency department: triggers, treatments, and outcomes. J Allergy Clin Immunol.

[20] Harduar-Morano L, Simon MR, Watkins S, Blackmore C (2011). A population-based epidemiologic study of emergency department visits for anaphylaxis in Florida. J Allergy Clin Immunol.

[21] Rudders SA, Banerji A, Corel B, Clark S, Camargo CA (2010). Multicenter study of repeat epinephrine treatments for food-related anaphylaxis. Pediatrics.

[22] Michelson KA, Monuteaux MC, Neuman MI (2015). Glucocorticoids and Hospital Length of Stay for Children with Anaphylaxis: A Retrospective Study. J Pediatr.

[23] Jerschow E, Lin RY, Scaperotti MM, McGinn AP (2014). Fatal anaphylaxis in the United States, 1999–2010: temporal patterns and demographic associations. J Allergy Clin Immunol.

[24] Lertnawapan R, Maek-a-nantawat W (2011). Anaphylaxis and biphasic phase in Thailand: 4-year observation. Allergol Int.

[25] Järvinen KM, Amalanayagam S, Shreffler WG, Noone S, Sicherer SH, Sampson HA (2009). Epinephrine treatment is infrequent and biphasic reactions are rare in food-induced reactions during oral food challenges in children. J Allergy Clin Immunol.

[26] Meng J, Rotiroti G, Burdett E, Lukawska JJ (2017). Anaphylaxis during general anaesthesia: experience from a drug allergy Centre in the UK. Acta Anaesthesiol Scand.

[27] Manivannan V, Campbell RL, Bellolio MF, Stead LG, Li JT, Decker WW (2009). Factors associated with repeated use of epinephrine for the treatment of anaphylaxis. Ann Allergy Asthma Immunol.

[28] Cardona V, Ferré-Ybarz L, Guilarte M, Moreno-Pérez N, Gómez-Galán C, Alcoceba-Borràs E (2017). Safety of adrenaline use in anaphylaxis: a multicentre register. Int Arch Allergy Immunol.

[29] Mobayed H, Ibrahim W, Al-Nesf M (2014). Delayed clavulanic acid-induced anaphylaxis in a patient undergoing bariatric surgery. Ann Allergy Asthma Immunol.

[30] Ehlayel M, Bener A, Abu Hazeima K, Al-Mesaifri F (2011). Camel milk is a safer choice than goat milk for feeding children with cow milk allergy. ISRN Allergy.

[31] Ehlayel MS, Hazeima KA, Al-Mesaifri F, Bener A (2011). Camel milk: an alternative for cow's milk allergy in children. Allergy Asthma Proc.

[32] Simons FER, Ardusso LRF, Bilò MB, El-Gamal YM, Ledford DK, Ring J (2011). World allergy organization guidelines for the assessment and Management of Anaphylaxis. World Allergy Organ J.

[33] Boyano-Martínez T, García-Ara C, Pedrosa M, Díaz-Pena JM, Quirce S (2009). Accidental allergic reactions in children allergic to cow's milk proteins. J Allergy Clin Immunol.

[34] Alshami A, Adeli M, Alyafei K, Nisar S (2018). Anaphylaxis presenting to the Pediatric Emergency Centers in Qatar. J Allergy Clin Immunol.

[35] Adeli M, Alyafei K, Chaudhry SI, Nisar S (2018). Incidence, Etiology and characteristics of adult onset anaphylaxis in Qatar. J Allergy Clin Immunol.

[36] Statistics Authority. Qatar Population Status 376 2012: Three Years After Launching the Population Policy, State of Qatar. https://www.ppc.gov.qa/en/Pages/default.aspx.

[37] Mahdavinia M, Fox SR, Smith BM, James C, Palmisano EL, Mohammed A (2017). Racial Differences in Food Allergy Phenotype and Health Care Utilization among US Children. J Allergy Clin Immunol Pract.

[38] Buka RJ, Crossman RJ, Melchior CL, Huissoon AP, Hackett S, Dorrian S (2015). Anaphylaxis and ethnicity: higher incidence in British south Asians. Allergy.

[39] Ganapathy S, Lwin Z, Ting DH, Goh LS, Chong SL (2016). Anaphylaxis in children: experience of 485 episodes in 1,272,482 patient attendances at a tertiary Paediatric emergency department from 2007 to 2014. Ann Acad Med Singap.

[40] Rebelo Gomes E, Geraldes L, Gaspar Â, Malheiro D, Cadinha S, Abreu C (2016). Hypersensitivity reactions to nonsteroidal anti-inflammatory drugs among adults: clinical features and risk factors for diagnosis confirmation. Int Arch Allergy Immunol.

[41] Orhan F, Canitez Y, Bakirtas A, Yilmaz O, Boz AB, Can D (2011). Anaphylaxis in Turkish children: a multi-Centre, retrospective, case study. Clin Exp Allergy.

[42] Al-Hammadi S, Zoubeidi T, Al-Maskari F (2011). Predictors of childhood food allergy: significance and implications. Asian Pac J Allergy Immunol.

[43] Jares EJ, Baena-Cagnani CE, Sanchez-Borges M, Ensina LF, Arias-Cruz A, Gomez M (2015). Drug-induced anaphylaxis in Latin American countries. J Allergy Clin Immunol Pract.

[44] Capps JA, Sharma V, Arkwright PD (2010). Prevalence, outcome and pre-hospital management of anaphylaxis by first aiders and paramedical ambulance staff in Manchester, UK. Resuscitation.

[45] Adatia A, Clarke AE, Yanishevsky Y, Ben-Shoshan M (2017). Sesame allergy: current perspectives. J Asthma Allergy.

[46] Irani C, Maalouly G, Germanos M, Kazma H (2011). Food allergy in Lebanon: is sesame seed the “middle eastern” peanut. World Allergy Organ J.

[47] Wetterer JK. Geographic spread of the samsum or sword ant, Pachycondyla (Brachyponera) sennaarensis (Hymenoptera: Formicidae). Myrmecological News. 2013;18:13–8.

[48] Al-Khalifa MS, Mashaly AM, Siddiqui MI, Al-Mekhlafi FA (2015). Samsum ant, Brachyponera sennaarensis (Formicidae: Ponerinae): distribution and abundance in Saudi Arabia. Saudi J Biol Sci.

[49] AlAnazi M, AlAshahrani M, AlSalamah M (2009). Black ant stings caused by Pachycondyla sennaarensis: a significant health hazard. Annals of Saudi Medicine.

[50] Thong BY, Leong KP, Chng HH (2005). Insect venom hypersensitivity: experience in a clinical immunology/allergy service in Singapore. Singap Med J.

[51] Nikbakhtzadeh MRAK, Tirgari S. Bioecology and chemical diversity of abdominal glands in the iranian samsum ant *Pachycondyla sennaarensis* (Formicidae: Ponerinae). J Venom Anim Toxins Incl Trop Dis. 2009;15(3):509–26.

[52] Dib G, Guerin B, Banks WA, Leynadier F (1995). Systemic reactions to the Samsum ant: An IgE-mediated hypersensitivity. Journal of Allergy and Clinical Immunology.

[53] Akbarzadeh MN K, Tirgari S, Abaei MR (2006). Medical Importnace of Fire Ant *Pachycondyla sennaarensis* (Hymenoptera: Formicidae) in Iranshahr and Sarbaz Counties, Southeastern of Iran. J Med Sci.

[54] Khan SA, Shelleh HH, Khan LA, Shah H (1999). Black fire ant (Solenopsis richteri) sting producing anaphylaxis: a report of 10 cases from Najran. Ann Saudi Med.

[55] Gelincik A, Demirtürk M, Yılmaz E, Ertek B, Erdogdu D, Çolakoğlu B (2013). Anaphylaxis in a tertiary adult allergy clinic: a retrospective review of 516 patients. Ann Allergy Asthma Immunol.

