# Supplementary material for: Anaphylaxis triggers in a large tertiary care hospital in Qatar: a retrospective study
Source: World Allergy Organ J. 2018 Sep 4;11(1):20. doi: 10.1186/s40413-018-0200-9 (PMC6122634; doi:10.1186/s40413-018-0200-9)
Supplement: Supplementary file 1 — Table S1. Anaphylactic patterns variation in relation to age, gender and nationality. a row percentage. Table S2. Symptoms of the study population. (DOCX 19 kb) [file 40413_2018_200_MOESM1_ESM.docx]

**Table S1.** Anaphylactic patterns variation in relation to age, gender and nationality

|  | | | | | | | | | | | |  |
| --- | --- | --- | --- | --- | --- | --- | --- | --- | --- | --- | --- | --- |
| Characteristics | | Anaphylaxis  N= 574  n(%)^a^ | | Food Anaphylaxis  N=316  n(%)^a^ | | Drug Anaphylaxis  N=103  n (%)^a^ | | Insects’ stings Anaphylaxis  N=161  n(%)^a^ | | Idiopathic  N=44  n(%)^a^ | |  |
| Age (Years) | |  | |  | |  | |  | |  | |  |
|  | < 10 Years | | 300 (77.9) | | 223 (74.3) | | 31 (10.3) | | 48 (16.1) | | 27 (9.0) | |
|  | 10 - 19 Y | | 109 (83.2) | | 51 (46.8) | | 15 (13.8) | | 40 (36.7) | | 7 (6.4) | |
|  | 20 - 55 Y | | 137 (86.7) | | 36 (27.1) | | 44 (33.1) | | 59 (44.0) | | 10 (7.5) | |
|  | > 55 Y | | 28 (87.5) | | 6 (21.4) | | 13 (46.4) | | 14 (50.0) | | 0 (0.0) | |
| *P-value* | |  | | *<0.001* | | *<0.001* | | *<0.001* | | *0.334* | |  |
| Gender | |  | |  | |  | |  | |  | |  |
|  | Male | | 315 (79.1) | | 199 (63.6) | | 47 (15.0) | | 63 (20.2) | | 29 (9.3) | |
|  | Female | | 259 (84.1) | | 117 (45.7) | | 56 (21.9) | | 98 (38.1) | | 15 (5.9) | |
| *P-value* | |  | | *<0.001* | | *0.033* | | *<0.001* | | *0.130* | |  |
| Nationality | |  | |  | |  | |  | |  | |  |
|  | Qatari | | 251 (79.9) | | 137 (55.2) | | 40 (16.1) | | 80 (32.3) | | 15 (6.0) | |
|  | Non-Qatari, Arab | | 162 (86.6) | | 86 (53.4) | | 32 (19.9) | | 39 (24.2) | | 14 (8.7) | |
|  | Asian | | 118 (83.1) | | 62 (52.5) | | 21 (17.8) | | 35 (29.7) | | 15 (12.7) | |
|  | Others | | 42 (67.7) | | 30 (73.2) | | 10 ( 23.8) | | 7 (17.1) | | 0 (0.0) | |
| *P-value* | |  | | *0.117* | | *0.589* | | *0.118* | | *0.333* | |  |

^a^ row percentage

| Table S2. Symptoms of the study population | | | | | |
| --- | --- | --- | --- | --- | --- |
| Symptoms | | **All subjects,**  **N= 1068, n (%)** | **Anaphylaxis**  **N = 574, n (%)** | **GAR**  **N = 132, n (%)** | **P-value** |
| Skin | | 564 (52.8) | 505 (87.9) | 15 (11.3) | 0.098 ^*^ |
|  | Rash | 434 (40.6) | 402 (70.0) | 11 (8.3) | 0.269 |
|  | Itching | 241 (22.5) | 222 (38.6) | 7 (5.3) | 0.835 |
|  | Urticaria | 217 (20.3) | 199 (34.6) | 8 (6.1) | 0.524 |
|  | Erythema | 173 (16.1) | 157 (27.3) | 9 (6.8) | **0.058** |
|  | Angioedema | 169 (15.8) | 157 (27.3) | 5 (3.7) | 0.893 |
|  | Local edema | 137 (12.8) | 120 (20.9) | 9 (6.8) | **0.019** ^*^ |
|  | Periorbital swelling | 78 (7.3) | 70 (12.1) | 3 (2.2) | 0.719 ^*^ |
|  | Fever | 35 (3.2) | 34 (5.9) | 1 (0.7) | 1.000 ^*^ |
|  | Conjunctivitis | 33 (3.1) | 30 (5.2) | 3 (2.2) | 0.085 |
| Respiratory | | 419 (39.2) | 397 (69.1) | 6 (4.5) | **<0.001** ^*^ |
|  | Dyspnea | 268 (25.1) | 253 (44.1) | 3 (2.2) | **0.011** |
|  | Cough | 138 (12.9) | 132 (22.9) | 3 (2.2) | 0.582 |
|  | Wheezing/Bronchospasm | 99 (9.2) | 95 (16.5) | 1 (0.7) | 0.337 ^*^ |
|  | Gasping | 69 (6.4) | 68 (11.8) | 0 (0.0) | 0.149 |
|  | Congested oropharynx/nose, +/- itching | 52 (4.8) | 48 (8.3) | 1 (0.7) | 1.000 ^*^ |
|  | Rhinitis | 38 (3.5) | 35 (6.1) | 3 (2.2) | 0.119 ^*^ |
|  | Hoarseness | 23 (2.1) | 23 (4.0) | 0 (0.0) | 1.000 ^*^ |
|  | Upper airway obstruction | 17 (1.5) | 17 (2.9) | 0 (0.0) | 1.000 ^*^ |
|  | Tachypnea | 17 (1.5) | 16 (2.7) | 0 (0.0) | 1.000 ^*^ |
|  | Stridor | 12 (1.1) | 12 (2.1) | 0 (0.0) | 1.000 ^*^ |
|  | Chest pain/tightness | 11 (1.0) | 9 (1.5) | 1 (0.7) | 0.254 ^*^ |
| Gastrointestinal | | 284 (26.5) | 273 (47.5) | 5 (3.7) | **0.054** |
|  | Vomiting | 186 (17.4) | 183 (31.8) | 2 (1.5) | **0.042** |
|  | Lips swelling, +/- itching | 78 (7.3) | 75 (13.1) | 3 (2.2) | 0.729 ^*^ |
|  | Abdominal pain | 53 (4.9) | 53 (9.2) | 0 (0.0) | 0.401 ^*^ |
|  | Tongue swelling, +/- itching | 25 (2.3) | 21 (3.6) | 0 (0.0) | 1.000 ^*^ |
|  | Diarrhea | 20 (1.8) | 20 (3.4) | 0 (0.0) | 1.000 ^*^ |
|  | Nausea | 11 (1.0) | 11 (1.9) | 0 (0.0) | 1.000 ^*^ |
|  | Swallowing difficulty | 6 (0.5) | 6 (1.0) | 0 (0.0) | 1.000 ^*^ |
| Cardiac | | 94 (8.8) | 91 (15.8) | 1 (0.7) | 0.333^*^ |
| Hypotension | | 123 (11.5 ) | 119 (20.7) | 2 (1.5) | 0.339 |
|  | Tachycardia | 35 (3.2) | 32 (5.5) | 1 (0.7) | 1.000 ^*^ |
|  | Syncope/loss of conscious | 22 (2.0) | 22 (3.8) | 0 (0.0) | 1.000 ^*^ |
|  | Cyanosis | 16 (1.4) | 16 (2.7) | 0 (0.0) | 1.000 ^*^ |
|  | Bradycardia | 5 (0.4) | 5 (0.8) | 0 (0.0) | 1.000 ^*^ |
|  | Crepitation | 4 (0.3) | 4 (0.6) | 0 (0.0) | 1.000 ^*^ |
| Nervous system | | 56 (5.2) | 51 (8.8) | 2 (1.5) | 0.686 |
|  | Dizziness | 39 (3.6) | 34 (5.9) | 2 (1.5) | 0.328 |
